# IgG4-Related Tubulointerstitial Nephritis Associated with Membranous Nephropathy in Two Patients: Remission after Administering a Combination of Steroid and Mizoribine

**DOI:** 10.1155/2014/678538

**Published:** 2014-06-19

**Authors:** Kana N. Miyata, Hiromi Kihira, Manabu Haneda, Yasuhide Nishio

**Affiliations:** ^1^Division of Nephrology and Hypertension, Harbor-UCLA Medical Center, 1124 W. Carson Street, Torrance, CA 90502, USA; ^2^Division of Nephrology, Department of Medicine, Tokyo Metropolitan Tama Medical Center, 2-8-29 Musashidai, Fuchu-shi, Tokyo 183-8524, Japan

## Abstract

We report two cases of Japanese men who presented with proteinuria, eosinophilia, hypocomplementemia, and high serum immunoglobulin G4 (IgG4) concentration and were diagnosed with membranous nephropathy associated with IgG4-related tubulointerstitial nephritis on renal biopsy. The typical renal lesions of IgG4-related disease are tubulointerstitial nephritis, which improves remarkably with steroid therapy, and occasional glomerular changes. In our two cases, renal biopsy revealed IgG4-positive immune complex deposits in glomeruli in a pattern of membranous nephropathy and concurrent tubulointerstitial nephritis with IgG4 plasma cells. In both cases, proteinuria persisted with initial prednisolone treatment and was resolved only after the addition of mizoribine. We report the first two cases in which the combination of prednisolone and mizoribine was effective for treating membranous nephropathy associated with IgG4-related tubulointerstitial nephritis.

## 1. Introduction

Recently, attention has been drawn to immunoglobulin G4- (IgG4-) related diseases (IgG4-RD), an autoimmune disease involving multiple organs, including the pancreas, kidney, salivary and lacrimal glands, lung, lymph nodes, and retroperitoneum [1]. It is characterized by elevated serum IgG4 concentration and abundant IgG4-positive plasma cell infiltration into the tissues. In addition, renal lesions are observed, indicating IgG4-related kidney disease (IgG4-RKD). The most common pattern observed on kidney biopsy is tubulointerstitial nephritis (IgG4-TIN); the typical presentation in this case includes a gradual decrease in kidney function with mild or no hematuria and minimal or absence of proteinuria [2]. Glomerular lesions have recently been described, including membranous nephropathy, membranoproliferative glomerulonephritis, and mesangial proliferative glomerulonephritis.

We herein present two cases of membranous nephropathy with concurrent IgG4-TIN, both of which were successfully treated by a combination of prednisolone and mizoribine.

## 2. Case Report

### 2.1. Case 1

The patient was a 69-year-old Japanese man who presented with anorexia and weight loss. Five months prior to admission, he had visited a local hospital complaining of lower abdominal pain; colonoscopy revealed early-stage colon cancer, and he underwent endoscopic resection. The patient's persistent abdominal pain, anorexia, and weight loss of >10% after two months prompted a concern for recurrence of malignancy; however, computed tomography (CT), magnetic resonance imaging, and positron emission tomography scans showed no abnormalities other than mildly enlarged supraclavicular and para-aortic lymph nodes. He was referred to our tertiary medical center for further evaluation. His past medical history included hypertension, dyslipidemia, and glaucoma for 10 years. Physical examination was normal, except for mild hypertension (143/80 mmHg). Laboratory findings are listed in Table 1. Urinalysis showed proteinuria (3+) quantitated at 1.4 g/day and blood (3+). Urine sediment showed microhematuria (>100/high-power field) with hyaline and granular casts. Blood tests showed low levels of complement, elevated absolute eosinophil count (5.462/*μ*L), and normal serum creatinine level 1.0 mg/dL (88.4 *μ*mol/L). Serum total IgG and IgG4 levels were elevated at 6989 mg/dL and 2750 mg/dL, respectively. An abdominal CT scan with contrast and kidney ultrasound showed no abnormalities and showed both kidneys at 9 cm.

**Figure 1:**
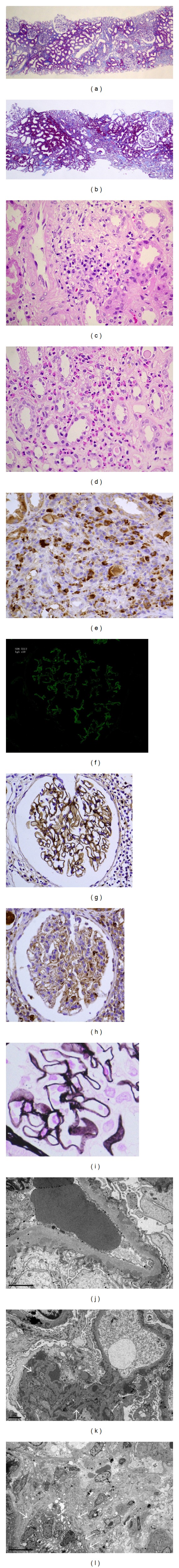
Representative microscopic histology. (a) Patchy infiltration of inflammatory cells and fibrosis in the interstitium with a clear border. Case 1. Masson trichrome stain, ×40. (b) Case 2. Masson trichrome stain, ×40. (c and d) Predominant infiltration of lymphocytes, plasma cells, and eosinophils into the interstitium. Case 1. H&E stain, ×400. (e) IgG4-positive plasma cells in the interstitium. Case 2. IgG4 immunoperoxidase stain, ×400. (f) Granular capillary wall staining for IgG. Case 1. Immunofluorescence, ×200. (g) IgG staining along the glomerular basement membrane (GBM). Case 1. IgG immunoperoxidase stain, ×400. (h) Weakly positive IgG4 staining along the GBM. Case 1. IgG4 immunoperoxidase stain, ×400. (i) Thickened GBM with spikes. Case 1. Periodic acid silver-methenamine stain, ×400. (j) Subepithelial electron-dense deposits. Case 1. ×20,000. (k) Subepithelial and mesangial electron-dense deposits. Case 2. ×10,000. (l) Scattered tubular basement membrane deposits. Case 2. ×4,000.

The renal biopsy tissue contained the cortex and medulla, with 27 glomeruli (Figure 1), and showed focal interstitial fibrosis accompanied by a patchy infiltration of mononuclear cells, eosinophils, and numerous IgG4-positive plasma cells (>10 per high-power field). The margins of the interstitial lesions were well defined. There were no glomerular scleroses or crescent formations. All glomeruli showed global thickening of the glomerular basement membrane (GBM) without duplication. There was no increase in mesangial cells, matrix, or endocapillary hypercellularity. Periodic acid silver-methenamine staining showed spikes. Immunofluorescence (IF) revealed a diffuse granular capillary wall staining of IgG and C1q without any extraglomerular deposits. IF indicated the absence of IgA, IgM, C3, and C4. Kappa and lambda staining were not performed. Immunoperoxidase stain showed weakly positive IgG4 staining along the GBM as well as interstitial inflammation, as indicated by IgG4-positive plasma cells. Electron microscopy showed subepithelial electron-dense deposits. Tubular basement membrane immune complex deposits were not seen.

Following the renal biopsy, the patient was diagnosed with IgG4-TIN and concurrent membranous nephropathy and was started on oral prednisolone 55 mg/day (0.9 mg/kg/day) with an immediate decrease in serum IgG levels and increase in serum complement. Proteinuria, however, persisted for over 2 months, fluctuating between 1.5 and 4 g/day, and oral mizoribine 100 mg/day was added. The patient's proteinuria finally trended down after initiation of mizoribine, reaching 0.4 g/day. Mizoribine was subsequently discontinued after the patient developed hepatotoxicity and was switched to azathioprine 25 mg/day. Currently, 4 years after the initial diagnosis, the patient is administered prednisolone 1 mg/day and azathioprine 25 mg/day, maintaining normal complement, normal serum creatinine level, and negative proteinuria. There were no extrarenal manifestations of IgG4-RD during this follow-up period.

### 2.2. Case 2

An 80-year-old Japanese man with a history of myocardial infarction, osteoarthritis, and 40-pack-year smoking was admitted to a local hospital with pancreatitis of unknown etiology 4 months prior to admission. One month after discharge, he developed leg edema on both legs and other features consistent with nephrotic syndrome, prompting referral to our medical center. Laboratory findings indicated proteinuria (2.73 g/day), eosinophilia (absolute eosinophil count 3.441/*μ*L), hypoalbuminemia, and hypocomplementemia (Table 1). Serum creatinine was 0.9 mg/dL (79.6 *μ*mol/L). Both IgG and IgG4 levels were elevated at 6350 mg/dL and 1350 mg/dL, respectively. Kidney ultrasound showed normal sized kidneys with small cysts. An abdominal CT scan with contrast showed a small amount of pleural effusion and ascites, without any lesions in either kidney.

Renal biopsy specimens showed interstitial infiltration of lymphocytes, plasma cells, and eosinophils, with accompanying interstitial fibrosis (Figure 1). Many of the plasma cells were positive for IgG4 staining (>10 per high-power field). All 11 glomeruli showed spikes along the glomerular capillary walls. Routine IF showed diffuse and global IgG, C3, and C1q depositions along the GBM and negative staining for IgA, IgM, and C4. Kappa and lambda staining were not performed. Granular deposition of IgG4 along the GBM was also seen on immunoperoxidase staining. Electron microscopy revealed subepithelial and mesangial electron-dense deposits and scattered tubular basement membrane deposits.

Oral prednisolone at an initial dose of 40 mg/day (0.6 mg/kg/day) reduced his serum complement and IgG levels, and proteinuria was resolved to some extent (0.3 g/day). Two months later, when the prednisolone dose was tapered, however, proteinuria recurred (2.6 g/day); mizoribine was added to his regimen, which resolved the proteinuria. He had another episode of proteinuria 10 months after the initiation of mizoribine, which was resolved with temporarily increased prednisolone dose. We followed up with this patient for 17 months after the initial diagnosis when he tested negative for proteinuria and had normal complement and serum creatinine levels, and, subsequently, we tapered the prednisolone dose to 10 mg/day with mizoribine 150 mg/day. There was no recurrence of pancreatitis or any other extrarenal manifestations of IgG4-RD during the follow-up period.

## 3. Discussion

While many studies have reported IgG4-RKD in the past decade, a proposal on diagnostic criteria has been only recently published [1, 13]. IgG4-TIN can be diagnosed on the basis of the histological features of plasma cell-rich TIN with >10 IgG4-positive plasma cells per high-power field in the most concentrated field and at least one other feature using imaging, serology, or other organ involvement [1]. Both our cases satisfied the criteria of histological features and serology (elevated serum total IgG and IgG4 levels). Imaging did not show any radiographic features of IgG4-TIN such as low-attenuation renal lesions or diffuse marked enlargement of the kidneys. Although other organ involvement was not apparent, mildly enlarged* supraclavicular and para-aortic* lymph nodes on CT in Case 1 and a recent history of pancreatitis of unknown etiology in Case 2 were assumed to be secondary to IgG4-RD.

It is unclear why most cases of IgG4-RKD show TIN, whereas some cases show concurrent glomerular lesions. Nishi et al. reviewed 37 cases of IgG4-RKD that presented with TIN, of which 10 cases (27%) had complicated glomerulonephritis, including mesangial proliferative glomerulonephritis, membranous nephropathy, membranoproliferative glomerulonephritis, and endocapillary proliferative glomerulonephritis [2]. Proteinuria was generally mild or absent in most solely IgG4-TIN cases; however, similar to our cases with concurrent glomerulonephritis, they had moderate proteinuria. Membranous nephropathy has been the most commonly observed glomerular lesion associated with IgG4-RD; recently, even membranous nephropathy without TIN but with other features of IgG4-RD has also been recognized as part of IgG4-RKD [9, 11, 12]. To date, 21 cases of membranous nephropathy as a manifestation of IgG4-RD (IgG4-related membranous nephropathy) have been reported (Table 2) [1, 3–16]. Interestingly, it is also recognized that primary membranous nephropathy is an IgG4-dominant disease, and it is important to distinguish IgG4-related membranous nephropathy from primary membranous nephropathy. The latest case reports showed the absence of circulating antiphospholipase A2 receptor antibodies in cases of membranous nephropathy with IgG4-RD, suggesting that the membranous nephropathy was not idiopathic but was likely secondary to IgG4-RD [10–12, 16].

Of the 15 cases in which the treatment and the outcome were reported, 13 cases were initially administered corticosteroid therapy based on the fact that typical IgG4-RD and IgG4-TIN improve remarkably with steroid therapy (Table 2). However, eventually, 3 cases required mycophenolate mofetil [11], 3 cases required rituximab [11, 12, 15], and 1 case was treated with cyclophosphamide [11]. During the follow-up period, 2 cases required maintenance hemodialysis [8, 15] and 1 case had a renal transplant [11]. Proteinuria in 7 cases remained at >1.5 g/day after treatment, though serum creatinine and proteinuria partially improved [4, 5, 11, 12]. Overall, the treatment was not completely successful in many of the IgG4-related membranous nephropathy cases.

In the two present cases, we initially failed to control proteinuria with steroids alone but observed a decrease to 0 g/day as well as maintenance of renal function after adding mizoribine. Mizoribine is an imidazole nucleoside with immunosuppressive activity, which inhibits T-cell and B-cell proliferation [17]. Its use has been associated with a low incidence of severe adverse reactions; in Japan, it is currently used widely for preventing rejection in renal transplantation and for the treatment of lupus nephritis, IgA nephropathy, and rheumatoid arthritis. Its immunosuppressive mechanism is similar to mycophenolate mofetil as an inhibitor of inosine 5′-monophosphate dehydrogenase, but it is recently proposed to have further unique properties; inhibition of renal macrophage accumulation and prevention of glomerulosclerosis and interstitial fibrosis in non-insulin-dependent diabetic kidneys [18], enhancement of glucocorticoid efficacy by binding to 14-3-3 protein and HSP60 in glomerular cells [19, 20], and better histologic outcome in IgA nephropathy in group of steroid plus mizoribine combination therapy compared to group of steroid monotherapy [21].

While IgG4-RD is generally known to have a favorable response to steroid treatment, a poor response in cases of proteinuria with steroid therapy suggests that, although TIN subsides with steroids, the membranous nephropathy may linger. In such cases, it is justified to treat patients with immunosuppressants.

In summary, we describe two cases of IgG4-TIN with concurrent membranous nephropathy that responded favorably to the combination of prednisolone and mizoribine. When renal function or proteinuria in IgG4-RKD does not respond well to steroid therapy, particularly when these conditions are associated with membranous nephropathy, the administration of immunosuppressants should be considered.

## Figures and Tables

**Table 1:** Laboratory findings.

	Case 1	Case 2
Urinalysis; protein/blood	3+/3+	2+/2+
Urinary protein (g/day)	1.4	2.73
Urinary *β*2MG (*μ*g/L)	11,080	858 (ref, 0–230)
Urinary NAG (U/L)	31.9	151.2 (ref, 0–11.5)
Creatinine (mg/dL)	1.0	0.9
CCr (mL/min)	92.4	49.1
BUN (mg/dL)	15.4	16.1
WBC (Eosinophil) (×10^9^/L) (%)	11.5 (47.5)	7.4 (46.5)
Hemoglobin (g/dL)	11.9	9.5
Hematocrit (%)	35.4	28.5
Total protein (g/dL)	9.3	7.6
Albumin (g/dL)	2.2	1.7
AST (U/L)	27	43
ALT (U/L)	15	33
ALP (U/L)	492	480
Bilirubin, total (mg/dL)	0.4	0.4
Amylase (U/L)	60	N/A
CRP (mg/dL)	1.7	0.4
CH50 (U/mL)	<5	<5 (ref, 25–48)
C3 (mg/dL)	39	21 (ref, 86–160)
C4 (mg/dL)	2.0	<2 (ref, 17–45)
Antinuclear antibody	1 : 320 (Homo/Spec)	<1 : 40
Anti-dsDNA antibody	Negative	Negative
Anti-Sm antibody	Negative	Negative
Anti-RNP antibody	Negative	16.0
Anti-SSA antibody	Negative	Negative
Anti-SSB antibody	Negative	N/A
ANCA	Negative	Negative
IgA (mg/dL)	183	386 (ref, 110–410)
IgM (mg/dL)	37	468 (ref, 83–190)
IgE (mg/dL)	413	1811 (ref, 0–202)
IgG (mg/dL)	6989	6350 (ref, 870–1700)
IgG1 (mg/dL)	1710	2460 (ref, 423–1080)
IgG2 (mg/dL)	2280	1740 (ref, 265–931)
IgG3 (mg/dL)	249	1100 (ref, 5–121)
IgG4 (mg/dL)	2750	1050 (ref, 4–108)
S-IL2 receptor (U/mL)	3912	3973 (ref, 190–650)
SPEP	Normal	Normal
Cryoglobulin	Negative	Negative

Note: Conversion factors for units: Creatinine in mg/dL to *μ*mol/L, ×88.4; CCr in mL/min to mL/s, ×0.01667; BUN in mg/dL to mmol/L, ×0.357; Hemoglobin, Total protein, and Albumin in g/dL to g/L, ×10; Bilirubin in mg/dL to mol/L, ×17.1; CRP in mg/dL to mg/L, ×10; IgA, IgM, IgE in mg/dL to mg/L, ×10; IgG, IgG1, IgG2, IgG3, and IgG4 in mg/dL to g/L, ×0.01. No conversion necessary for C3, C4 in mg/dL and g/L.

Abbreviations and definitions: *β*2MG, *β*2 microglobulin; NAG, N-acetyl-beta-D-glucosaminidase; CCr, creatinine clearance; WBC, white blood cell; AST, aspartate aminotransferase; ALT, alanine aminotransferase; ALP, alkaline phosphatase; CRP, C-reactive protein; ANCA, antineutrophil cytoplasmic antibody; IgA, immunoglobulin A; S-IL2 receptor, soluble interleukin-2 receptor; SPEP, serum protein electrophoresis; ref, reference range.

**Table 2:** Treatment and the effect of IgG4-related membranous nephropathy reported in previous studies.

Case	References, Year	Age	Gender	IgG4/IgG	TIN	S-Cr (mg/dL)		U-pro (g/day)		f/u (months)	Treatment
		(years)		(mg/dL)		Pre Tx	Post Tx	Pre Tx	Post Tx		
1	Uchiyama-Tanaka et al. 2004 [3]	64	Male	665/5410	yes	4.5	1.5	1.5	0.1	2	mPSL
2	Watson et al. 2006 [4]	67	Male	2125/7070	yes	3.4	2.4	2.4	2.5	7	PSL
3	Saeki et al. 2009 [5], Saeki et al. 2010 [6], Saeki et al. 2013 [7]	83	Male	924/3144	yes	1.48	1.22	2.3	1.6	4	PSL
4	Saida et al. 2010 [8], Saeki et al. 2010 [6], Saeki et al. 2013 [7]	78	Male	1860/3731	yes	6.17	HD	1.38	HD	NA	PSL
5	Palmisano et al. 2010 [9]	68	Male	NA	no	1.2	NA	1.5	0.45	NA	ACEI, statins
6	Raissian et al. 2011 [1]	75	Male	NA	yes	6.6	2.5	16	NA	1.1	PSL
7	Raissian et al. 2011 [1], Fervenza et al. 2011 [10], Alexander et al. 2013 [11]	67	Female	531/1581	yes	1.2	1.3	4	0.2	7	PSL
8	Cravedi et al. 2011 [12]	54	Male	730/2022	no	1.15	NA	7.3	8	11	PSL, Rituximab
9	Kawano et al. 2011 [13]	NA	NA	NA	yes	NA	NA	NA	NA	NA	NA
10	Yamaguchi et al. 2012 [14]	60	Female	142/1706	yes	1.05	NA	14	NA	NA	NA
11	Yamaguchi et al. 2012 [14]	64	Male	260/4292	yes	1.4	NA	0.67	NA	NA	NA
12	Jindal et al. 2012 [15]	72	Male	750/1511	yes	4.7	HD	15	HD	NA	PSL, Rituximab
13	Alexander et al. 2013 [11]	75	Male	NA	yes	6.6	2.5	16	3.1	6	PSL
14	Alexander et al. 2013 [11]	53	Male	NA	no	0.8	0.8	16	3.1	46	PSL, MMF
15	Alexander et al. 2013 [11]	50	Male	NA	no	1.3	1.3	3.5	1.5	20	ACEI/ARB, Rituximab, MMF
16	Alexander et al. 2013 [11]	46	Female	NA	yes	NA	Transplant	Nephrotic	0	184	none
17	Alexander et al. 2013 [11]	67	Male	NA	yes	1.8	1.4	4.5	0.7	7	PSL, Cy, ACEI
18	Alexander et al. 2013 [11]	65	Female	NA	yes	0.7	0.8	8.5	2.6	4	PSL, MMF
19	Alexander et al. 2013 [11]	64	Male	NA	yes	3.4	NA	1.7	NA	NA	NA
20	Alexander et al. 2013 [11]	34	Male	NA	no	1.6	NA	12.4	NA	NA	NA
21	Li et al. 2014 [16]	55	Male	1926/5305	yes	0.8	NA	5.5	0.2	20	PSL
22	Miyata et al. 2014 (Case 1)	69	Male	2750/6989	yes	1.0	1.17	1.4	0	48	PSL, MZR, AZA
23	Miyata et al. 2014 (Case 2)	80	Male	1050/6350	yes	0.9	0.78	2.73	0	17	PSL, MZR

Abbreviations: ACEI, angiotensin-converting enzyme inhibitor; ARB, angiotensin receptor blocker; AZA, Azathioprine; Cy, cyclophosphamide; f/u, follow up; HD, hemodialysis; mPSL, methylprednisolone; MMF, Mycophenolate mofetil; MZR, mizoribine; PSL, prednisone/prednisolone; S-Cr, NA, not applicable; serum creatinine; TIN, tubulointerstitial nephritis; U-Pro, urine protein.
